# Modification of Cellulosic Materials with Boron-Nitrogen Compounds

**DOI:** 10.3390/polym15132788

**Published:** 2023-06-23

**Authors:** Irina Stepina, Aleksey Zhukov, Sofia Bazhenova

**Affiliations:** Department of Building Materials Science, National Research Moscow State University of Civil Engineering Yaroslavskoe sh. 26, 129337 Moscow, Russia; lj211@yandex.ru (A.Z.); sofia.bazhenova@gmail.com (S.B.)

**Keywords:** wood, cellulose, boron-nitrogen compounds, modification, bio-protection, fire-protection, analytical optimization

## Abstract

Wood fiber and its products are modified to increase fire and bio-resistance. The best results are achieved by using modifiers that enter into chemical interaction with the hydroxylated substrate, forming the organic matrix of the materials. The purpose of the research described in the article was to study the possibility of using boron-nitrogen compounds to modify cellulose and cellulose-containing materials to improve the performance, bio- and fire-protective properties of construction materials, as well as to optimize the consumption of boron-nitrogen compounds. As a result of the research, it was found that the boron-nitrogen compounds used in the compositions developed here chemically interact with hydroxyl groups at the C^6^-atom of cellulose. The chemical interaction of boron-nitrogen compounds with cellulose is an inter-crystalline process occurring without destruction of the crystal structure of the substrate since the modifier molecules bind with the more accessible hydroxyl groups of the amorphous regions of cellulose. Thus, surface modification with boron-nitrogen compounds does not result in accelerated aging of cellulose-containing materials and loss of strength but, on the contrary, increases the durability of wooden structures.

## 1. Introduction

For synthetic building materials, foamed plastics, and materials based on natural fibers, there are very acute problems with reducing their flammability increasing their biological resistance, including resistance to putrefaction. The need to improve fire and bio-resistance applies even more to heat-isolation materials made of natural fibers, such as cellulose fibers, and materials made of expanded plastics [1,2,3].

The highly porous structure of these materials makes them vulnerable to various types of biological attacks. The first very presence of porosity implies that these pores are filled with a vapor-air mixture. The moisture vapor contained in the air is adsorbed on the surface of the pores as well as the fibers within the equilibrium state by a monomolecular or polymolecular adsorption mechanism. If the matrix of the material consists of components prone to corrosion (rotting or fungal attack, among others), it becomes possible to destroy products, release harmful substances and spores, etc. [4,5,6]. These factors reduce both the environmental safety of structures and the durability of materials, and consequently, the durability of structures [7,8,9].

The presence of oxygen in the “atmosphere” of pores also adversely affects the rot resistance of the material; moreover, oxygen as an oxidant accelerates the processes of rot damage and, in the case of fire exposure, creates favorable conditions for combustion [10,11,12]. Because open communicating porosity prevails in fibrous materials and it is impossible to isolate internal volumes from environmental contacts, the main way to improve the resistance of high-porous fibrous materials, including those based on cellulose, is to modify the properties of the matrix, i.e., the fibrous component [13,14].

The modification of wood fiber and its products is carried out in two directions: wood preservation (chemical protection) and wood modification (modifying protection). While chemical protection is performed with preservatives, modification is done by activating chemical components present in wood cell walls using high temperatures [15]. Popular wood preservatives such as creosote, pentachlorophenol, and copper naphthenate prolong the service life of industrial wood products but require additional money and time for subsequent disposal of treated wood and restoration of contaminated areas [16,17,18]. Water-borne chemicals were introduced into the market in the 1950s [19]. Their carrier is cost-effective, safe, and normally provides a clean surface on the treated wood [20]. Preserved wood with water-borne chemicals can be painted post-treatment and can also be used in a wider range of applications, such as utility poles, residential lumber, and timber, as well as for the protection of wood composites (American Wood Protection Association) [21]. Water-borne treatments include arsenical, non-arsenical copper, and non-metal preservatives. Numerous studies were conducted on the fungicide, bactericide, insecticide, algaecide, and moldicide performances of water-borne chemicals [22,23,24,25].

Among water-soluble wood preservatives, borates are the most effective [26,27]. In addition, they have low toxicity to mammals but are quite effective against wood-destroying insects and fungi [28]. Borates have excellent performance in indoor applications, but their fixation problem constrains their use outdoors. Although the fixation of borate compounds has been extensively studied [29,30,31,32,33], it is still recommended by AWPA (American Wood Protection Association) only for interior applications [34]. This is due to the fact that boron-containing preservatives include trivalent boron compounds. The ester bonds formed between trivalent boron compounds and hydroxyl groups of cellulose are hydrolytically unstable, which is explained by the presence of a vacant atomic orbital in the outer electron layer of the boron atom. Due to the nucleophilic attack, water molecules form a complex with the boron-containing cellulose ether, which decomposes and leads to the hydrolysis of the ester bond. It is logical to assume that if tetravalent boron-atom compounds, which have no free atomic orbitals, are used to modify the wood surface, the ester bond between the modifier and the substrate can be stabilized. In this connection, it seems reasonable to use boron-nitrogen compounds for wood modification in molecules in which the coordination of the unshared electron pair of the nitrogen atom with the free atomic orbital of the boron atom (boron-amine coordination) leads to stabilization of the ester-bond between modifier molecules and components of the ligno-carbon complex, which eliminates nucleophilic attack by water molecules and provides reliable fixation of the modifier in the substrate [35,36].

If we specify the field of studies with natural fibers and, in particular, with wood fiber, cellulose is the main component (about 50% by mass), but in view of its crystalline structure, it is the least reactive component of the lignocarbohydrate complex (LCC) of wood. Therefore, initially, the possibility of chemical interaction of the obtained boron-nitrogen modifiers with reactive groups of LCC was studied exactly on cellulose under “soft” modification conditions, and then the obtained experimental data were checked on wood [37,38]. The aim of the studies was to obtain reactive quadruple-coordinated boron-nitrogen compounds from commercially available boric acid and amino alcohols (mono- and diethanolamine) and to study the possibility of using the obtained boron-nitrogen compounds for modifying cellulose and cellulose-bearing materials to improve the performance, bio-, and fire-protective properties of natural polymers.

## 2. Materials and Methods

The modified materials were cellulose and pine sawdust mechanically ground to particle sizes no larger than 1 mm, which were modified at room temperature by immersion for 3 h in 50% aqueous solutions containing four-coordinated boron-nitrogen compounds: monoethanolamine(N→B)-trihydroxyborate ((HO)_3_B←NH_2_-(CH_2_)_2_-OH) and diethanolamine(N→B)-trihydroxyborate ((HO)_3_B←NH-((CH_2_)_2_-OH)_2_) (hereinafter compositions 1 and 2, respectively). The boron-nitrogen compounds were obtained by mixing boric acid with mono- and diethanolamine in an equimolar ratio with water [39]. To remove excess modifiers, cellulose, and pine sawdust samples were extracted with distilled water, then air-dried at room temperature to a constant weight.

The obtained modified cellulose samples were studied by X-ray photoelectron spectroscopy (XPSS) on an XSAM-800 spectrophotometer made by Kratos (UK). Registration of spectra was carried out at room temperature; the depth of the studied surface was 10 nm. The IR spectra of the samples were recorded on a Magna-750 FT-IR spectrometer by Nicolet (Madison, Wisconsin USA) in the mid-infrared region of 4000–400 cm^−1^ with a spectral resolution of 2 cm^−1^. To prepare the samples, cellulose and wood were dissolved with KBr (about 2 mg of the substance per 100 mg of KBr) and pressed into tablets, from which the spectra were obtained. The spectrum of pure KBr (which always has a small amount of adsorbed water) was subtracted from the obtained spectra. X-ray diffraction analysis was performed on a Scientific model ARL X’TRA Termo diffractometer by Termo Electron SA (Ecublens, Switzerland) using radiation λ[CuKα] = 1.5418 Å and a Ni filter with a rotating sample.

The experimental study of cellulose modification was conducted on the basis of analytical optimization [40,41]. The results of the experiment were processed using the program “Statistics”. The technique of analytical optimization consists of conducting a multifactor active experiment according to D-optimal plans. Regression equations were formed based on the results, and mathematical analysis apparatus is applied to these equations. As a result, the degree of influence of each significant factor on the result was established. Testing of statistical hypotheses was carried out using Student’s and Fisher’s criteria. The confidence interval was calculated using the student’s criterion values. The conditions of the experiment are presented in Table 1. The area of fungus surface lesions in the samples was taken as a response function.

To test the biological stability of the modified wood, samples of native wood (50 × 50 × 10 mm) were processed for 3 h in 10%, 30%, and 50% aqueous solutions of composition 1 and composition 2.

Following that, in accordance with GOST 9.048, the surface of the wooden samples was infected with a suspension containing 1–2 million spores per mL of the fungi Aspergillus niger van Tieghem, Aspergillus terreus Thom, Aureobasidium pullulans (de Bary) Arnaud, Paecilomyces variori Bainier, Penicillium funiculosum Thom, PenicillAdditionally, spores of the wood-destroying fungi Serpula lacrimans BKM-465 and Antrodia sinuosa BKM F-1741 were introduced into the suspension.

Unmodified wood samples were used as a control. The samples infected with the above-mentioned fungi suspension were placed in an open Petri dish in a desiccator and kept under optimal conditions for fungi growth: 27–28 °C and 98% humidity for 28 days. Intermediate examinations of the samples (visually and by microcopying under a magnification of 60×) were performed every 14 days. At the end of the tests, the stage of fungus development was evaluated on scores according to the 5-point system (GOST 9.048–89), proportional to the surface area of the samples affected by fungus.

## 3. Results and Discussion

Unmodified control wood samples were overgrown with fungi on 80–85% of the surface; intensive development of all types of fungal test cultures and sporulation was observed on them.

**Figure 1:**
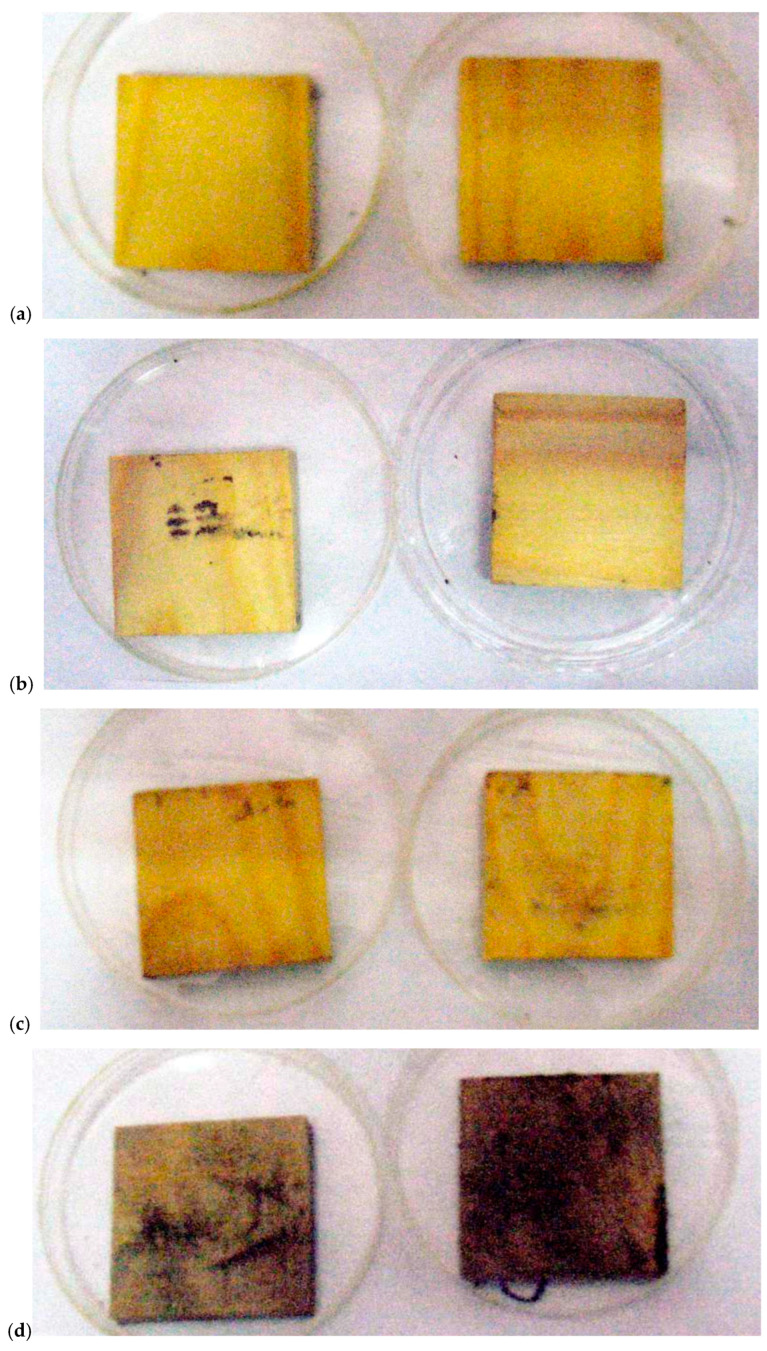
Appearance of samples after testing according to GOST 9.048–89: (**a**) 50% compositions of modifiers; (**b**) 30% compositions of modifiers; (**c**) 10% compositions of modifiers; (**d**)-control (unmodified wood samples).

Wood samples modified with 10% aqueous solutions of compounds 1 and 2 revealed abundant growth of mycelium from mold and wood-destroying fungi on the surface of the samples. Samples of wood modified with 30% aqueous solutions of modifiers are more fungus-resistant. Surface modification of wood samples in 50% aqueous solutions of compositions 1 and 2 provides 100% biostability of treated wood with respect to mold and wood-destroying fungi (Figure 1 and Table 2).

After processing the results of the experiment, checking statistical hypotheses, and sifting out non-significant coefficients, the following basic regression equation was obtained (confidence interval is 2.1), establishing the relationship between biostability (the value of the inverse area of the fungus surface of samples) (Y) and the factors to be varied:

У = 80.0 + 5.8X_1_ + 11.0X_2_ + 4.8X_1_X_2_ − 1.4X_1_^2^ (1)

where X_1_—Particle size of crushed cellulose materials; X_2_—Modifier concentration, which intervals are presented in Table 1.

The greatest influence on the result was concentration of modifier (coefficient at X_2_ equal to 11.0). The influence of the particle size has a much smaller effect, and at values of the sizes approaching the maximum limit, the intensity of growth of biostability decreases (coefficients at X_1_ and X_2_ are equal to 5.8 and minus 1.4, respectively). Optimal for treatment is the particle size of cellulose material, which is 0.9–1.0 mm.

**Figure 2:**
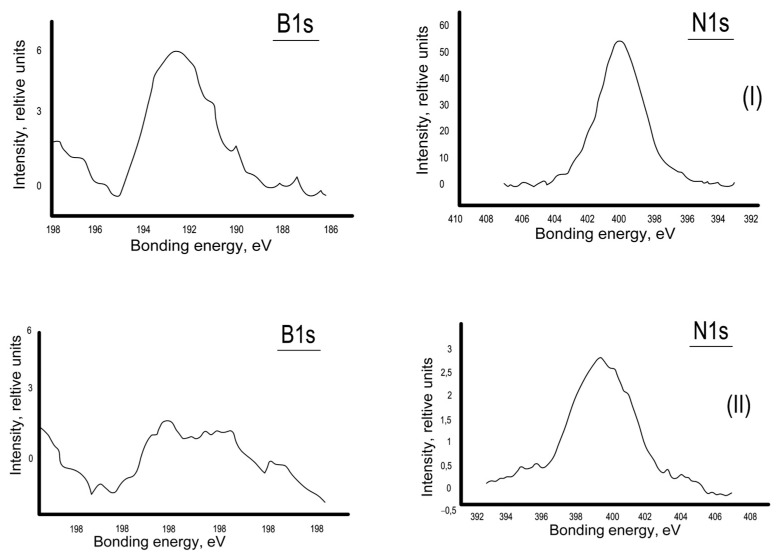
Photoelectron spectra of B 1s and N 1s samples of cellulose modified with compositions 1 (**I**) and 2 (**II**).

Studies of the composition of the samples treated with modifying compositions show that the sample of cellulose (Table 3 and Figure 2) modified with composition 2 contains less boron than the sample of cellulose modified with composition 1. This fact agrees with the experimental data obtained by ^11^B NMR spectroscopy [39]; according to these data, composition 2 is a mixture of two substances: a boron-containing ether with a three-coordinated boron atom (diethylaminborate) and a four-coordinated boron-nitrogen compound (diethanolamine(N→B)-trihydroxyborate) in a mass ratio of 3:1, respectively, of which only the latter compound forms hydrolytically stable bonds with reactive groups in cellulose (cellulose). Molecules of the three-coordinated ether (diethylaminborate) do not form hydrolytically stable bonds with alcohol groups of cellulose; boron-containing cellulose ethers obtained as a result of the transesterification reaction are hydrolyzed in the process of extraction with distilled water, and molecules containing the three-coordinated boron atoms were washed off the substrate surface with water.

The values of binding energies of 1 s-electrons with the nuclei of boron and nitrogen atoms included in the cellulose samples modified by compositions 1 and 2 and the known values of binding energies of atoms B and N from literature sources [42] are compared in Table 4. As a result, the obtained B1s bond energy result did not agree with any of the literature sources, values which were calculated for the three-coordinated boron atoms. The fixed chemical shift of the 1s bond energy of the electron with the boron atom nucleus confirms that in our case the boron atom and hence the nitrogen atom in the modified cellulose samples are four-coordinated, which serves to confirm the formation of a donor-acceptor bond between them [42].

**Figure 3:**
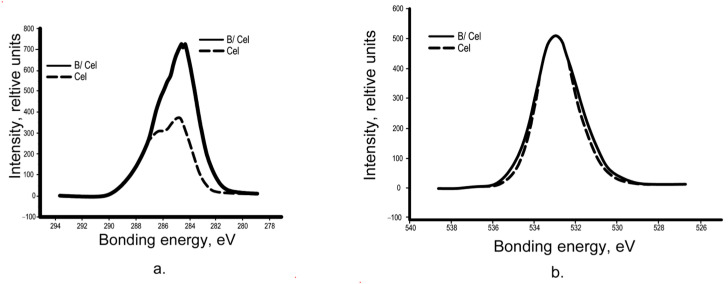
Photoelectron spectra of cellulose: (**a**) modified; (**b**) unmodified cellulose.

The appearance of new asymmetry in the spectral peaks characteristic of oxygen and carbon atoms in the X-ray photoelectron spectra of modified samples as compared with similar peaks of unmodified cellulose provides evidence of chemical interaction between the obtained modifiers and reactive groups of cellulose. When asymmetry manifests, new chemical connections between the atoms O and B and C and O have formed (Figure 3).

The fact that in spite of long extraction with distilled water, boron and nitrogen atoms remain in the composition of modified cellulose samples also confirms that four-coordinated boron compounds are reacting with the alcohol groups of cellulose. As known from the literature, this is possible only if the formed ester bonds between modifier molecules and the OH-groups of cellulose are hydrolytically stable, i.e., the boron atom included in the composition of modifiers has no free atomic orbitals, which rules out hydrolysis.

Thus, based on experimental data obtained by X-ray photoelectron spectroscopy, it has been established that the obtained modifiers—Compositions 1 and 2 containing the four-coordinated boron compounds monoethanolamine(N→B)-trihydroxyborate ((HO)_3_B←NH_2_-(CH_2_)_2_-OH) and diethanolamine(N→B)-trihydroxyborate ((HO)_3_B←NH-((CH_2_)_2_-OH)_2_)—chemically interact with the hydroxyl groups of cellulose. For a more detailed study of the character of the interaction of the modifier molecules with wood components, a detailed analysis of the IR spectra of cellulose and wood vein modified with compositions 1 and 2 in comparison with control samples was carried out.

To study the nature of the interaction of wood components with compositions based on four-coordinated boron-nitrogen compounds, a detailed analysis of modified cellulose and wood by FTIR spectroscopy was carried out. Mechanically crushed cellulose was used as cellulose samples, and crushed pine sawdust was used as wood samples (the particle size in both cases did not exceed 1 mm).

It should be noted that cellulose and wood modification were carried out by two fundamentally different methods. The first one implies a one-stage treatment of cellulose with compositions based on water and boron-nitrogen compounds. Monoethanolamine(N→B)-trihydroxyborate ((HO)_3_B←NH_2_-(CH_2_)_2_-OH) and diethanolamine(N→B)-trihydroxyborate ((HO)_3_B←NH-((CH_2_)_2_-OH)_2_) were used in this case in 50% aqueous solutions.

The second method of modification involved treating cellulose and wood in two stages with composition components (solutions of boric acid and aminoalcohols) in different sequences. The concentration of boric acid solution was 5% by weight (this is its maximum solubility at 20 °C). The concentration of mono- and diethanolamine solutions was 25% by mass (the same amount of alcohol was used to obtain 50% aqueous solutions of boron-nitrogen compounds).

The modification process involved immersion in modifier solutions for three hours at room temperature. The excess modifiers were removed by extraction with distilled water. Further samples were dried at room temperature to maintain a constant weight.

**Figure 4:**
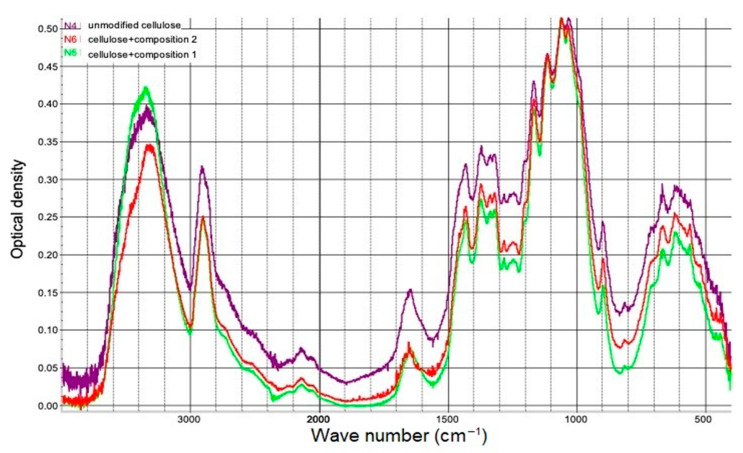
IR spectra of cellulose samples.

The results of IR-spectra registration of the obtained samples are shown in Figure 4.

Authors should discuss the results and how they can be interpreted from the perspective of previous studies and of the working hypotheses. The findings and their implications should be discussed in the broadest context possible. Future research directions may also be highlighted.

Unfortunately, the newly formed bonds N→B and B-O, whose presence in the composition of the modified cellulose was proved by the XPSS method, absorb in the same regions as the C-C, C-O bonds. At the same time, the absorption bands overlap in the spectra. However, we may discuss variations in the strength of the corresponding peaks that occur when cellulose is chemically modified, as seen in the spectra in Figure 4. Changes in the intensity of corresponding peaks upon chemical modification of cellulose were evaluated relative to the internal standard that was taken as the peak with a frequency of 897 cm^−1^ corresponding to valent vibrations of the glucopyranose ring of cellulose. The characteristic frequency of the N→B coordination bond is the frequency of 3100 cm^−1^ [44]. Unfortunately, it is impossible to identify a peak with such a frequency in the IR spectrum of modified cellulose because of overlapping absorption bands. The absorption at 1200–1100 cm^−1^ characterizes the valence vibrations of N→B in the tetrahedron [44]. The intensity of peaks relative to the internal standard within the indicated frequencies in the IR spectra of modified cellulose is much higher than that of unmodified cellulose (Figure 4). This is a confirmation of the grafting of boron-nitrogen compounds onto the substrate surface. The 1340 cm^−1^ absorption band characterizes the B-O-C, C-O-C, and C-C bonds [44]. Much more intense absorption bands of this frequency in the IR spectra of modified cellulose are another confirmation of the covalent grafting of boron-nitrogen compounds.

The broad absorption band in the 3700–3100 cm^−1^ region of the IR spectrum of cellulose is associated with valent vibrations of hydroxyl groups involved in hydrogen bonds. It is known that the low-frequency region of the ν_OH_ band characterizes hydroxyls involved in stronger hydrogen bonds (intramolecular) and the high-frequency region weaker ones (intermolecular) [45]. In the spectra of modified cellulose, a shift in the ν_OH_ absorption band is observed, namely, an increase in absorption on the high frequency side, especially in the spectrum of cellulose modified with composition 2, Figure 4. According to literature sources [46], this is associated with an increase in the proportion of hydroxyls involved in weak hydrogen bonds. Probably, the modification reactions in both cases proceed predominantly along the primary, sterically more accessible hydroxyl group located near the C_6_-atom of the glucopyranose cycle.

Valent vibrations of C-H bonds of methylene and methine groups of cellulose are proposed in the region of 3000–2800 cm^−1^ [47]. In the spectra of modified cellulose (Figure 4), these valence vibrations are superimposed on the absorption of CH_2_ groups, which are part of the four-coordinated boron-nitrogen compounds. This leads to an increase in the intensity of absorption bands with a frequency of 2900 cm^−1^ in the spectra in Figure 4, since the modification causes the grafting of derivatives containing additional CH_2_ groups onto the surface of cellulose samples.

In the region of 1650 cm^−1^ the adsorbed water molecules are absorbed. With increasing water content, the absorption band maximum shifts slightly towards higher wave numbers [47], which is also observed in the spectra of modified samples (Figure 4). The grafting of OH acid hydroxyls and polar amino groups, which are part of the tetracoordinated amine borates, increases the polarity of the substrate, which contributes to retaining more adsorption water at the surface of the modified cellulose samples due to the hydrogen bond system.

The absorption bands (crystallinity band) of 1431 cm^−1^ and 900 cm^−1^ (amorphous band) in the spectrum of original cellulose, Figure 4, correspond to the scissor vibrations of the methylene group and vibrations of the C1 atom and four surrounding atoms in the spectra of β-glycoside structures. When the degree of crystallinity of cellulose increases as a result of mechanical or chemical modification, the intensity of the 1431 cm^−1^ band increases and the 900 cm^−1^ band decreases [47], which is observed in the IR spectra of modified samples, i.e., when modifying cellulose with compositions based on four-coordinated compounds and water, the degree of crystallinity and ordering of modified cellulose samples increase [48].

The 1060 cm^−1^ absorption band in the spectrum of cellulose is attributed to the valence vibration of the C-O bond in the HC_3_-OH group. Band 1030 cm^−1^ correlates with valent vibrations of the C-O bond in the primary alcohol group in various conformations. In the spectra of cellulose modified by both Composition 1 and Composition 2, Figure 4, a decrease in the intensity of the 1030 cm^−1^ frequency peak compared to the 1060 cm^−1^ frequency peak indicates that hydroxyl groups at C_6_ of the cellulose macromolecule (the primary alcohol group) are involved in the modification [47].

**Figure 5:**
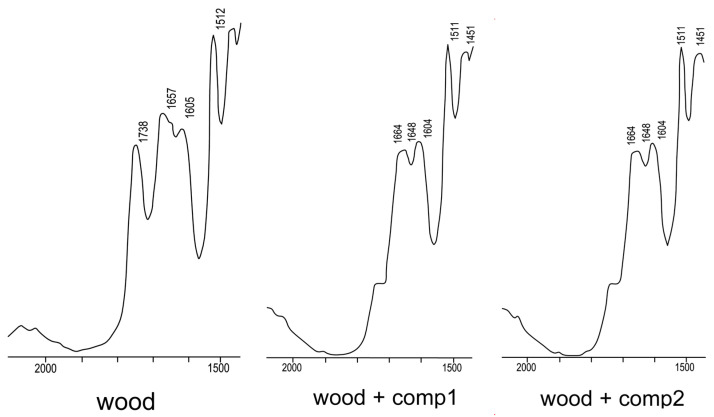
Fragments of IR spectra of unmodified and modified wood.

Comparative analysis of infrared spectra of original wood and wood modified by compositions 1 and 2 showed that there are no qualitative differences in the examined spectra, except for the absorption band of 1730 cm^−1^, corresponding to valent vibrations of carboxyl groups of lignin, Figure 5 [47]. A significant decrease in the intensity of the 1738 cm^−1^ band in the spectra of wood samples modified by Composition 1 and Composition 2, Figure 5, indicates that the obtained tetracoordinated bosonic compounds are capable of chemically interacting with carboxyl groups of lignin.

Thus, the experimental data obtained by FTIR spectroscopy confirms the fact of the chemical interaction of the obtained four-coordinated boron-nitrogen compounds with cellulose and wood lignin, with the formation of hydrolytically stable ethers containing four-coordinated boron and nitrogen atoms on the surface of the modified materials. The modification does not destroy intramolecular bonds and increases the degree of crystallinity (orderliness) of the substrate, indicating that the modification process proceeds under mild conditions (“soft” modification). A probable scheme of interaction of monoethanolamine(N→B)-trihydroxyborate and diethanolamine(N→B)-trihydroxyborate with cellulose can be represented as follows. 
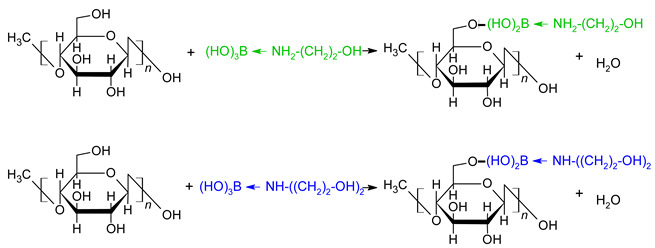


At the next stage, the possibility of obtaining four-coordinated boron-nitrogen compounds directly on the surface of cellulose by consecutive modification with aqueous solutions of initial substances (boric acid and aminoalcohols) in different sequences was studied: in the sequence (a), there are 1-boric acid (aq); 2-monoethanolamine (aq); in the sequence (b), there are 1-monoethanolamine (aq); 2-boric acid (aq); in the sequence (c), there are 1. Boric acid (aq); 2. diethanolamine (aq); and in the sequence (d), there are 1-diethanolamine (aq); and 2-boric acid (aq).

**Figure 6:**
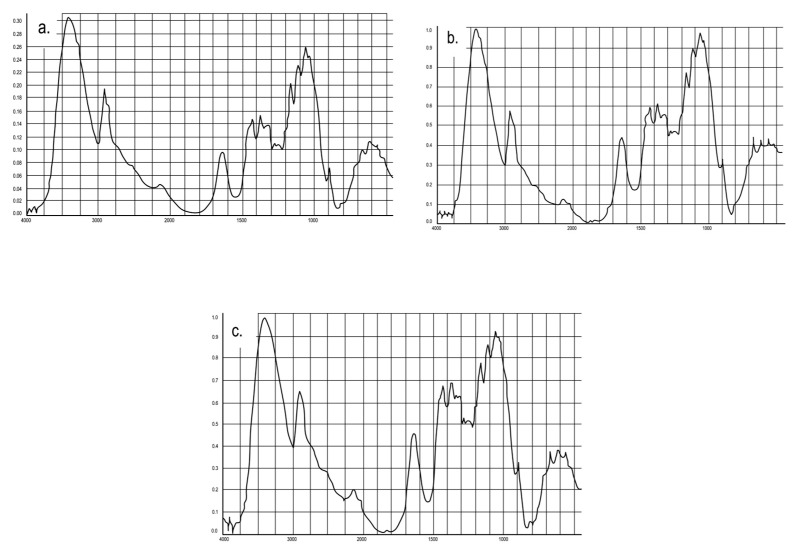
IR spectra: (**a**) cellulose + boric acid (solution); (**b**) cellulose + monoethanolamine (solution); (**c**) cellulose + diethanolamine (solution).

The IR spectra of cellulose modified with aqueous solutions of boric acid and aminoalcohols are shown in Figure 6. It is known from literature [47] that the intensity of the valence vibration band 2900 cm^−1^ (ν_C-H_) is used as an internal standard to estimate the intensity of a broad absorption band in the range of 3600–3100 cm^−1^ characterizing the valence vibrations of hydroxogroups involved in hydrogen bonds (provided that the number of C-H valence bonds in the substrate structure is not changed as a result of modification). The ratio of the optical densities of the 3340 cm1/2910 cm^−1^ bands in the samples of unmodified cellulose and cellulose modified with boric acid solution is 1.25 and 1.63, respectively. This indicates that the modification of cellulose with boric acid solution increases the number of hydroxyls on the substrate surface due to the grafting of acid hydroxyl groups. Based on O’Connor’s work [47], crystallinity indices (ratio of optical densities D1430/D897) were calculated in the studied samples, whose values are 1.35 and 2.18 for native and borated cellulose, respectively. The obtained results indicate the presence of chemical interactions between boric acid molecules and alcohol groups in cellulose [49].

In the IR spectra of cellulose modified with mono- and diethanolamine solutions, Figure 6, are sharply increased in comparison with unmodified cellulose, Figure 4, the intensity of 1640 cm^−1^ frequency absorption bands, which correspond to absorption of adsorbed water molecules and, according to [47], strain vibrations of N-H bonds of primary (at 1600 cm^−1^) and secondary (at 1650 cm^−1^) amines. The crystallinity indices for cellulose modified with mono- and diethanolamine solutions are 1.78 and 2.02, respectively, i.e., in both cases, the ordering of modified samples is higher than that of unmodified samples (1.35). An increased degree of crystallinity indicates the presence of chemical interaction; otherwise, some changes in the IR spectra of cellulose treated with solutions of aminoalcohols could be considered a consequence of the dissolution of cellulose fibers in organic alcohols; however, in this case, the degree of crystallinity of cellulose would decrease dramatically [50].

It is interesting to note that the absorption intensity of the 1200 cm^−1^ band, which characterizes symmetrical valence vibrations of the glycosidic bond in the cellulose macromolecule [47], decreases in the spectra of modified samples in Figure 6 (cellulose + monoethanolamine, cellulose + diethanolamine, cellulose + boric acid) compared to the original cellulose (Figure 4). This is probably due to the partial hydrolysis of cellulose during its modification with aqueous solutions of boric acid and aminoalcohols, followed by extraction with distilled water.

As a result, cellulose’s reactive groups can be chemically interacted with by boric acid, monoethanolamines, and diethanolamines, allowing for the gradual alteration of cellulose.

It should be emphasized that because all IR spectra are qualitatively similar, analyzing the spectra of stage-by-stage changed cellulose samples poses certain challenges. The broad absorption band that corresponds to the valence vibrations of hydroxyl groups present in hydrogen bonds, the range between 3700 and 3100 cm^−1^, is known to be the region of the cellulose spectrum where chemical alteration of the material causes the greatest modifications. In [45], the following values were proposed to characterize the ν_OH_ band: the symmetry index (ratio of the left (a) and right (c) parts of the OH-group absorption band width measured from the middle of the perpendicular drawn through the maximum) and the position of the ν_OH_ absorption maximum. The degree of substitution during the chemical alteration of cellulose is also indicated by a drop in the symmetry index [45]. Additionally, the absorption band shift in the 1630–1655 cm^−1^ area as well as the change in this band’s intensity in comparison to the nearby peaks were examined, along with the crystallinity index (ratio of optical densities D1430/D897) for each sample. The strength of the absorption band at 1630–1655 cm−1 implies that the modified cellulose contains -NH_2_ and =NH amino groups [47].

Table 5 shows some characteristics of the broad absorption band in the range 3700–3100 cm^−1^ (symmetry index, max ν_OH_), and the 1630–1655 cm^−1^ absorption band. Crystallinity index values for: 1. unmodified cellulose samples; 2. cellulose samples modified with solutions of boric acid (ba) and amino alcohols (monoethanolamine, mea, and diethanolamine, dea); and 3. cellulose samples modified in stages with aqueous solutions of boric acid and amino alcohols in different sequences.

Table 5 shown that when cellulose is modified with solutions of boric acid and aminoalcohols, as well as stage by stage, the maximum absorption of OH-groups shifts towards higher wave numbers, the symmetry index decreases, and the crystallinity index increases. This indicates changes in the system of hydrogen bonds acting between the hydroxyls of cellulose, in particular a decrease in the number of hydroxyl groups bound by less strong hydrogen bonds, i.e., the presence of chemical interaction between the molecules of all modifiers and the alcoholic groups of cellulose. Based on the value of the symmetry index, the degree of substitution of OH groups in cellulose decreases in the following sequence: cellulose (cell) + ba > cell + ba + dea > cell + ba + mea > cell + mea + ba > cell + mea = cell + dea; the graft density during sequential modification is higher if the first stage of cellulose is esterified with boric acid. Moreover, when borated cellulose is modified with aminoalcohol solutions, the degree of substitution decreases (the symmetry index increases). This is probably due to the hydrolysis of ether bonds formed between boric acid and the hydroxyl groups of cellulose and the washout of boric acid in the process of modification with aqueous solutions of aminoalcohols and subsequent extraction.

The degree of substitution is higher (the symmetry index is higher) when altering first with aminoalcohols and then second with boric acid than when modifying alone with aminoalcohols. This, we presume, is because boric acid molecules autonomously engage with the hydroxyl groups of cellulose rather than the reactive groups of mono- and diethanolamines. This type of interaction between the modifiers and the substrate prevents the production of boron-nitrogen compounds with four covalent bonds on the surface of the cellulose. This is confirmed by the fact that the modification of cellulose with solutions of aminoalcohols dramatically increases the relative intensity of the absorption band in the region of 1630–1655 cm^−1^, which confirms the presence of amino groups in the modified samples. In the spectra of cellulose modified with compositions 1 and 2, the intensity of this band is low and is due to deformation vibrations of adsorbed water molecules, which absorb in the same region (the band of the valent vibrations of the N-H bond of the tetrahedral nitrogen atom is located in the areas 1200 and 3100 cm^−1^).

The analysis of infrared spectra of wood samples was modified stage-by-stage: Aqueous solutions of boric acid and aminoalcohols present certain difficulties due to a large number of absorption bands, many of which overlap each other; therefore, it was decided to analyze changes in intensity of only one peak with a frequency of 1730 cm^−1^ characterizing valent vibrations of carboxyl groups of lignin. To compare changes in the intensity of the 1730 cm^−1^ absorption band in the spectra of wood modified stepwise by solutions of boric acid, mono- and diethanolamines, relative to control (unmodified wood), the 1600 cm^−1^ absorption band, which corresponds to the valent vibrations of the lignin aromatic ring, was used as an internal standard. The values of D1600 and D1730 are given in Table 6.

Table 6 demonstrates that the relative strength of the 1738 cm^−1^ absorption band largely remains unchanged when the surface of the wood is modified with boric acid solution. The intensity of the 1738 cm^−1^ absorption band considerably lowers when solutions of amino alcohols are used to modify the wood surface (Table 6). This suggests that the hydroxyl groups of lignin and amino alcohols interact chemically. The following pattern can be seen in the IR spectra of wood samples that have undergone successive modifications: the studied band’s intensity is higher in samples that have first been esterified with boric acid and then treated with solutions of amino alcohols (Table 6), and the band intensity is lower in samples that have only been modified with monoethanolamine. It is interesting to note that in the spectra of wood samples modified with compositions 1 and 2, the relative intensity of the 1730 cm^−1^ absorption band is higher than in the spectra of samples modified with aqueous monoethanolamine solution, staged in combinations of boric acid-moethanolamine, monoethanolamine-boric acid, and diethanolamine solution, and staged in combinations of boric acid-diethanolamine and diethanolamine-boric acid modifiers, respectively. It can be assumed that aminoalcohols are more active in esterification reactions than the four coordinated boron-nitrogen compounds.

Thus, based on the experimental data obtained by FTIR spectroscopy, the following conclusions were drawn: when cellulose and wood are modified with compositions based on tetracoordinated boron-nitrogen compounds and water, hydrolytically stable esters containing tetracoordinated boron and nitrogen atoms are formed on the substrate surface. The modifiers interact with the hydroxyl groups of cellulose located at the sixth carbon atom of the glucopyranose ring and with the carboxyl groups of lignin. When modifying the cellulose surface in a stepwise manner, boric acid and amino alcohols interact independently of each other. The sequential treatment does not lead to the formation of hydrolytically stable tetracoordinated boron-nitrogen compounds on the cellulose surface.

**Figure 7:**
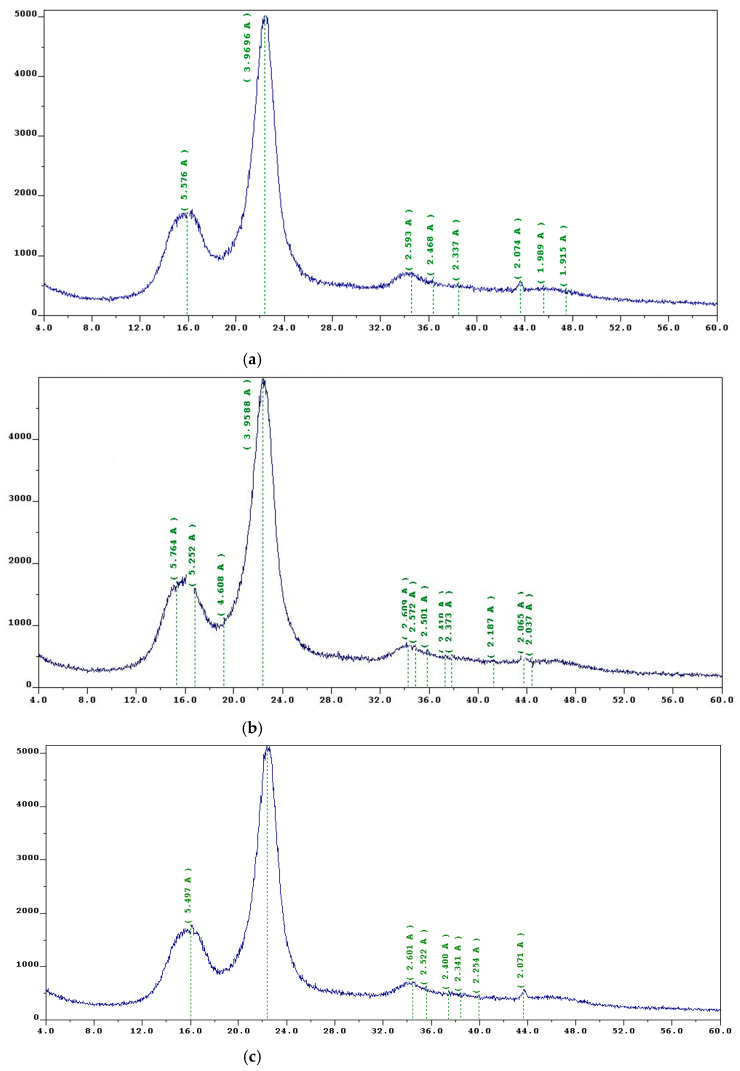
(**a**) X-ray diffraction of unmodified cellulose; (**b**) X-ray radiograph of cellulose modified with compound 1; (**c**) X-ray radiograph of cellulose modified with compound 2.

The obtained modifiers—four-coordinated boron-nitrogen compounds—chemically interact with reactive groups of the lignocarbohydrate complex of wood, resulting in the formation of hydrolytically stable bonds. However, if in the process of chemical modification there is a destruction of the crystal structure of cellulose, the main structural component of wood. It accelerates the aging of the wood and, as a result, causes a rapid loss of strength and collapse of wooden constructions. In this context, X-ray diffraction analysis was used to examine the structure of cellulose that had been changed using four-coordinated boron-nitrogen compounds. The results are shown in Figure 7.

There are peaks on every X-ray radiograph that correspond to the crystalline portion of cellulose’s X-ray scattering, as well as an amorphous halo that is a smooth portion of the curve with maximum intensity at 2° (Bregg angle) 19°. All cellulose samples contain two crystal maximums at the equator of the radiographs in the range of scattering angles up to 34°, with the first one resulting from X-ray reflection from planes 101 and 10-1 (216°) and the second one originating from plane 002 (222.2°). The reflection of X-rays from planes parallel to the plane of fixation of the findings causes a reflex on the X-ray meridian (234.3°) [51].

As can be seen from the radiographs, the blurred and superimposed 101 and 10-1 peaks on the radiograph of unmodified cellulose (Figure 7a) turn into clearly distinguishable reflexes on the radiographs in Figure 7b,c. This fact, as well as the increase in the degree of crystallinity (FC) of cellulose samples modified by compositions 1 and 2, indicates the ordering of cellulose structure in the process of its modification with four-coordinated boron-nitrogen compounds. The increase in IC of modified cellulose samples indicates that the chemical interaction of cellulose with reactive groups of amine borates is an intercrystalline process that proceeds without destruction of the crystal structure of cellulose since the molecules of modifiers react with more accessible hydroxyl groups in amorphous regions of cellulose [51].

Based on the experimental data obtained, the following conclusions were drawn: when modifying cellulose and, respectively, wood with the developed compositions, the crystalline structure of cellulose was not destroyed. Consequently, surface modification with boron-nitrogen compounds does not lead to accelerated aging of cellulose-bearing materials and rapid loss of strength, but rather increases the durability of wooden structures. Electron scanning microscopy data also testify to the increasing orderliness of cellulose and wood.

## 4. Conclusions

The biostability of material samples containing cellulose depends to a greater extent on the concentration of modifying solutions. Both types of solutions have approximately the same efficiency, and solution-treated samples react to the concentration of modifying solutions approximately the same. The optimal size for treatment is a particle size of 0.9–1.0 mm for cellulosic material.

It is possible to conclude from experimental results from X-ray photoelectron spectroscopy, FTIR spectroscopy, X-ray structural analysis, and scanning electron spectroscopy that the boron-nitrogen compounds used in the compositions developed here chemically interact with hydroxyl groups at the C6-atom of cellulose. The chemical interaction of boron-nitrogen compounds with cellulose is an inter-crystalline process occurring without destruction of the crystal structure of the substrate since the modifier molecules bind with the more accessible hydroxyl groups of the amorphous regions of cellulose. Thus, surface modification with boron-nitrogen compounds does not result in accelerated aging of cellulose-containing materials and loss of strength but, on the contrary, increases the durability of wooden structures.

## Figures and Tables

**Table 1:** Experimental conditions.

Name of the Factor	Symbol, Xi	The Average Value of the Factor, $\overset{–}{X}$i	Variation Interval, ΔXi	Factor Values at Levels	
				−1	+1
Particle size of crushed cellulose materials, mm	X_1_	0.8	0.2	0.6	1.0
Modifier concentration, %	X_2_	30	20	10	50

**Table 2:** Results of the assessment of bioproofness of wood according to GOST 9.048–89.

Concentration Compositions, %	Post-Test Appearance	Score	Bioresistance, %
***Composition 1***			
10	mycelium overgrowth on the surface	3	50
30	superficial mycelial growth	2	70
50	visually and under the microscope are clean	0	100
***Composition 2***			
10	mycelium overgrowth on the surface	3	50
30	superficial mycelial growth	2	70
50	visually and under the microscope are clean	0	100
***Control***			
	80–85% of the surface is overgrown with mushrooms	5	0

**Table 3:** Results of quantitative analysis obtained by the XPSS method of unmodified cellulose and tested samples.

Elmt	Cellulose + Composition 1	Cellulose + Composition 2	Control
	c, at.%	c, at.%	c, at.%
C 1s	65.90	74.28	70.85
O 1s	33.08	25.49	29.15
N 1s	0.54	0.23	-
B 1s	0.48	0.19	-

**Table 4:** Obtained values of binding energies by X-ray photoelectron spectroscopy (XPSS).

	B 1s	N 1s	Handbook [42]	NIST B1s [43]
Cellulose + Composition 1	192.6	400.8	BN: 190.7, 398.3 B_2_O_3_: 193.4 BOH: 193.2	190.5, 398.2 193.6, 533.2 H_3_BO_3_: 193.4
Cellulose + Composition 2	191.9	399.1		

**Table 5:** Comparative characteristics of IR spectra of modified and unmodified cellulose.

Samples	Symmetry Index (*a/в*)	Max ν_oн_, cm^−1^	Crystallinity Index (*D*_1430_/*D*_900_)	Strip~1630–1655 cm^−1^ *(intensity in relation to neighboring peaks)*
unmodified cellulose	1	3340	1.35	1647
cellulose + ba	0.53	3427	2.18	1641 (increase)
cellulose + mea	0.69	3424	1.78	1636 (increase)
cellulose + dea	0.69	3395	2.02	1644 (increase)
cellulose + ba + mea	0.62	3408	1.82	1643(increase)
cellulose a + ba + dea	0.57	3416	1.94	1636 (increase)
cellulose + mea + ba	0.64	3428	1.85	1640 (increase)
cellulose + dea + ba	0.68	3413	1.93	1636 (increase)

**Table 6:** Values of the relative intensity of the 1730 cm1 band (D1600/D1730) in the spectra of modified and original wood.

Samples	Relative Intensity *D*_1600_/*D*_1730_
unmodified wood	1.04
wood + ba	1.14
wood + mea	4.02
wood + dea	2.02
wood + ba + mea	3.27
wood ba + dea	1.82
wood + mea + ba	4.07
wood + dea + ba	2.05

## Data Availability

Not applicable.
